# Calculation of additional load and deformation of the receiving well enclosure structure caused by shield tunneling

**DOI:** 10.1371/journal.pone.0297912

**Published:** 2024-04-04

**Authors:** Li xin, Li-Guang Chen, Tong Li, Yi-lin Yu, Bo Wu

**Affiliations:** 1 China Construction Municipal Engineering Corporation Limited, Beijing, China; 2 China Construction First Group Corporation Limited, Beijing, China; Southwest Jiaotong University, CHINA

## Abstract

The bulkhead additional thrust during shield tunneling, the force of friction between shield and soil, and the additional grouting pressure can cause additional stress in the surrounding soil, thereby disturbing existing buildings and structures. However, few studies focused on the disturbance situation when the shield tunneling machine approaches the receiving well. If the additional stress and deformation of the receiving well are too excessive, it could result in the collapse of the receiving well. Based on the two-stage method, this study derived the calculation formula of the additional stress and deformation of the receiving well enclosure structure caused by shield tunneling. Taking a shield machine receiving engineering as the context, this study established a numerical simulation model and compared theoretical calculation, the results of numerical simulation model and on-site monitoring data. Finally, the additional stress of the receiving well is analyzed. The research findings demonstrate that the theoretical prediction results, numerical simulation calculation results, and on-site monitoring data exhibit relatively small calculation errors, which validated the applicability of the theoretical prediction formula and numerical simulation model. As the distance between the shield machine and the receiving well decreases, the disturbance to the receiving well increases sharply. When the distance between the cutter head and the receiving well is less than three times the shield length, it is crucial to enhance the deformation monitoring of the receiving well. The primary factors affecting the additional load and deformation of the receiving well enclosure structure are the force of friction between shield and soil and the additional thrust of the cutterhead. The disturbance caused by the additional grouting pressure on the enclosure structure can be ignored.

## Introduction

With the rapid pace of urbanization, urban traffic congestion has reached alarming levels. The subway, one of the widely utilized public transportation systems, offers numerous benefits such as high passenger capacity, punctuality, and minimal land occupation. Consequently, an increasing number of subway lines have been constructed in recent years [1–3]. The shield tunneling method is predominantly employed for subway construction in urban areas. Given the high concentration of existing buildings, the disturbance to the surrounding environment caused by shield tunneling has always been a hot topic of concern for scholars [4–6].

Extensive research has been conducted on the environmental disturbances resulting from shield tunneling. Deng, et al. [7] derived a prediction formula for surface settlement and deformation of existing pipelines caused by shield tunneling along curved paths, analyzing the associated disturbances. Wei and Jiang [8] presents a simplified analytical solution for calculating the longitudinal displacement of existing tunnels that are subjected to adjacent surcharge loading. Based on the Boussinesq solution, the distribution of the additional load matrix caused by the surface surcharge on the existing tunnel was obtained. Liu, et al. [9] proposed a simplified analysis method to evaluate the response of existing tunnels to shield tunneling. Lou, et al. [10] established a three-dimensional finite element model for studying the effects of double shield tunnel excavation on displacement and internal forces beneath existing buildings. Zhong-Hui, et al. [11] investigated the environmental impact of ultra-large diameter Earth Pressure Balance (EPB) shield tunneling beneath airports in soft soil. Zhe, et al. [12] examined the monitoring data before and after the shield passed Fengqi Bridge in Hangzhou Metro Line 2 to analyze the associated effects.

Overall, many scholars have studied the disturbances caused by shield tunneling on existing tunnels, buildings, and bridges. Various research methods have been employed, including numerical simulation, theoretical calculations, and on-site measurements [13–16]. In urban shield tunnel engineering, the starting, receiving, and turning processes of the shield machine often take place in receiving or starting wells, particularly in deep-buried or urban areas. When the shield machine approaches the receiving well enclosure structure, it breaks the structure to complete the receiving work through the thrust of the cutterhead. The combined action of the bulkhead additional thrust, the frictional force between the shield and soil, and the grouting pressure induces additional stress and deformation in the enclosure structure. If the shield machine causes excessive deformation of the receiving well when approaching the receiving well, it may lead to displacement and detachment of the support structure, and even cause instability and damage to the receiving well. However, limited research exists on the stress and deformation of the receiving well caused by shield tunneling. Thus, it is crucial to clarify the additional stress and deformation generated near the receiving well enclosure structure and analyze the impact of various construction factors on it. This will provide valuable insights for shield machine receiving engineering.

This study begins by employing a two-stage method to derive a prediction formula for quantifying the additional stress and deformation induced in the receiving well enclosure structure by shield tunneling. Subsequently, using a shield machine receiving engineering project as the basis, a numerical simulation model is established. Theoretical calculations, results from the numerical simulation model, and on-site monitoring data are then compared to assess the effectiveness and suitability of the theoretical prediction formula and numerical simulation model. The findings demonstrate that the proposed formula and model accurately capture the phenomenon. Finally, the study analyzes the additional stress and deformation in the receiving well enclosure structure resulting from various factors associated with shield tunneling. The research outcomes provide valuable theoretical insights and reference experiences for shield machine receiving engineering.

## Theoretical basis

In this paper, we focus solely on the additional deformation of the receiving shaft enclosure induced by construction loads during shield tunneling. The primary construction loads during shield tunneling encompass the following factors: the additional thrust generated by the cutter plate, friction within the shield shell, supplementary grouting pressure, and extra loads. For the purpose of this study, we will disregard any additional deformations resulting from non-construction loads in the surrounding soil environment. Examples of such loads include the self-weight of the shield machine, the solidification and expansion of slurry, and the loads associated with the tube sheet.

Additionally, we will not account for environmental factors that may influence the additional deformation of the receiving shaft, such as groundwater seepage, the solidification process of synchronized grouting slurry, and the consolidation of the surrounding soil. The calculation domain considered in this study is a semi-infinite elastic space. To simplify the computational burden posed by non-uniformly distributed soil layers, we employ the layer method, a technique widely established in previous research [17]. This method homogenizes the non-uniform distribution of soil layers by treating them as equivalent uniform layers.

Hirai [17] originally formulated the theory of the equivalent layered method, and it has since been a standard approach in handling non-uniform soil layer distributions. Building upon previous studies [18–20], it is reasonable to adopt the layer method to address the non-uniform distribution of soil layers. This approach greatly simplifies the computational complexities associated with non-uniform soil layer distributions. The conversion formula for this method is as follows:

$$H_{je}=\{\begin{array}{l} {\left[\frac{E_{j}(1-{\mu_{n}}^{2})}{E_{n}(1-{\mu_{j}}^{2})}\right]}^{1/3}H_{j},E_{j}>E_{n}\\ \left(\frac{3}{4}+\frac{1}{4}{\left[\frac{E_{j}(1-{\mu_{n}}^{2})}{E_{n}(1-{\mu_{j}}^{2})}\right]}^{1/3}\right)H_{j},E_{j}\leq E_{n} \end{array}$$ (1)

where *j* is the number of the soil layer, 1≤*j*≤*n*; *E*_*j*_ is the elastic modulus of layer soil *j*; *μ*_*j*_ is the Poisson’s ratio of layer soil *j*; *H*_*j*_ is the actual thickness of *j*th layer; *H*_*je*_ is the equivalent thickness of *j*th layer; *En* is the elastic modulus for *n*th layer (base soil layer); *μ*_*n*_ is the Poisson’s ratio for nth layer (base soil layer).

### Mindlin solution

The Mindlin solution [21] is employed to calculate the stress and displacement at various points within a semi-infinite elastic body resulting from horizontal or vertical loads applied at specific locations. The Mindlin calculation formula offers a concise mechanical model and requires fewer parameters for computation. It finds wide application in addressing disturbances to the surrounding environment caused by soil excavation, including the calculation of surface settlement induced by shield tunneling and the assessment of stress and deformation in neighboring existing buildings affected by shield tunneling. Numerous scholars have extensively investigated these research questions using Mindlin solutions in conjunction with on-site observations, numerical simulations, or centrifugal experiments, thereby establishing the reliability of Mindlin solutions. Based on the Mindlin solution, this study derives a formula to calculate the additional stress experienced by the receiving well enclosure structure resulting from shield tunneling.

**Fig 1:**
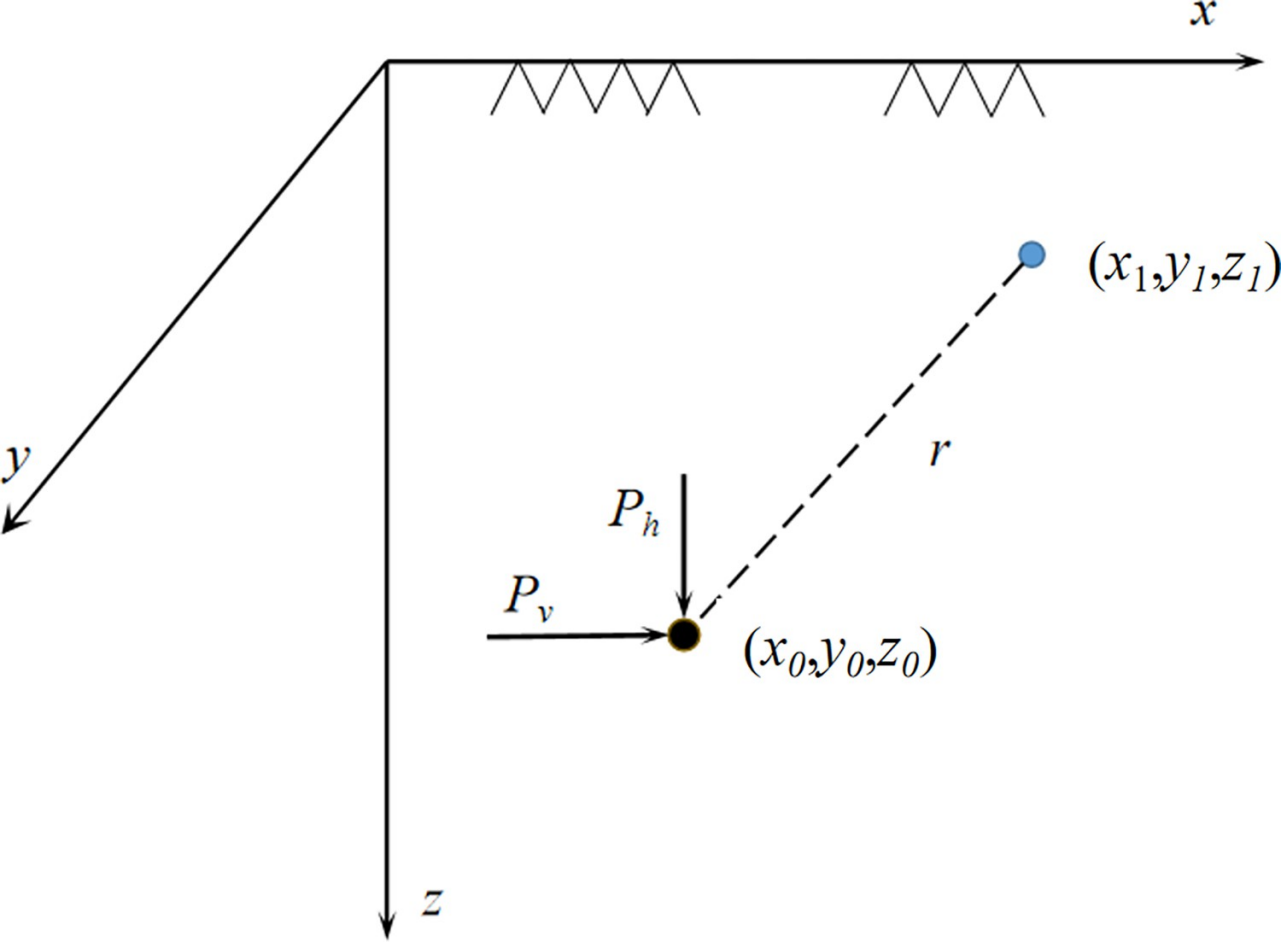
Mindlin solution diagram.

Fig 1 illustrates the calculation model of the Mindlin solution, where the coordinates of the load application point are denoted as (*x*_*0*_,*y*_*0*_,*z*_*0*_). Within the semi-infinite body, any point’s coordinates are represented as (*x*_*1*_,*y*_*1*_,*z*_*1*_). At the load application point, both a horizontal load *P*_*v*_ and a vertical load *P*_*h*_ are applied in the *x*-direction. At a specific point (*x*_*0*_,*y*_*0*_,*z*_*0*_), when a vertical unit load *P*_*h*_ along the *z*-direction is exerted, the calculation formula for determining the resulting additional stress at the point (*x*_*1*_,*y*_*1*_,*z*_*1*_) is expressed by Eqs (1) to (3):

$$\begin{array}{l} \sigma_{x1}=\frac{P_{V}}{8\pi (1-\mu )}\{\frac{\left(1-2\mu )(z_{0}-z_{1}\right)}{R_{1}^{3}}-\frac{3{\left(x_{0}-x_{1}\right)}^{2}(z_{0}-z_{1})}{R_{1}^{5}}+\frac{\left(1-2\mu )[3(z_{0}-z_{1})-4\mu (z_{0}+z_{1})\right]}{R_{2}^{3}}-\\ \;\frac{3(3-4\mu ){\left(x_{0}-x_{1}\right)}^{2}(z_{0}-z_{1})-6z_{1}(z_{0}+z_{1})[z_{0}(1-2\mu )-2\mu z_{1}]}{R_{2}^{5}}-\frac{30z{\left(x_{0}-x_{1}\right)}^{2}z_{0}(z_{0}+z_{1})}{R_{2}^{7}}-\\ \;\frac{4(1-\mu )(1-2\mu )}{R_{2}(R_{2}+z_{0}+z_{1})}(1-\frac{{\left(x_{0}-x_{1}\right)}^{2}}{R_{2}(R_{2}+z_{0}+z_{1})}-\frac{{\left(x_{0}-x_{1}\right)}^{2}}{R_{2}^{2}})\} \end{array}$$ (2)

$$\begin{array}{l} \sigma_{y1}=\frac{P_{V}}{8\pi (1-\mu )}\{\frac{\left(1-2\mu )(z_{0}-z_{1}\right)}{R_{1}^{3}}-\frac{3{\left(y_{0}-y_{1}\right)}^{2}(z_{0}-z_{1})}{R_{1}^{5}}+\frac{\left(1-2\mu )[3(z_{0}-z_{1})-4\mu (z_{0}+z_{1})\right]}{R_{2}^{3}}-\\ \;\frac{3(3-4\mu ){\left(y_{0}-y_{1}\right)}^{2}(z_{0}-z_{1})-6z(z_{0}+z_{1})[z_{1}(1-2\mu )-2\mu z_{1}]}{R_{2}^{5}}-\\ \;\frac{30z_{1}{\left(y_{0}-y_{1}\right)}^{2}z_{0}(z_{0}+z_{1})}{R_{2}^{7}}-\frac{4(1-\mu )(1-2\mu )}{R_{2}(R_{2}+z_{0}+z_{1})}(1-\frac{{\left(y_{0}-y_{1}\right)}^{2}}{R_{2}(R_{2}+z_{0}+z_{1})}-\frac{{\left(y_{0}-y_{1}\right)}^{2}}{R_{2}^{2}})\} \end{array}$$ (3)

$$\begin{array}{l} \sigma_{z1}=\frac{P_{V}}{8\pi (1-\mu )}\{-\frac{\left(1-2\mu )(z_{0}-z_{1}\right)}{R_{1}^{3}}-\frac{3{\left(z_{0}-z_{1}\right)}^{3}}{R_{1}^{5}}+\frac{\left(1-2\mu )(z_{0}-z_{1}\right)}{R_{2}^{3}}-\\ \;\frac{3(3-4\mu )z_{0}{\left(z_{0}+z_{1}\right)}^{2}-3z_{1}(z_{0}+z_{1})(5z_{0}-z_{1})}{R_{2}^{5}}-\frac{30z_{1}z_{0}{\left(z_{0}+z_{1}\right)}^{3}}{R_{2}^{7}}\} \end{array}$$ (4)

In this formula: *μ* is Poisson’s ratio of soil; *R*_*1*_ = [(*x*_*0*_*-x*_*1*_)^2^+(*y*_*0*_*-y*_*1*_)^2^+(*z*_*0*_*-z*_*1*_)^2^]^1/2^; *R*_*2*_ = [(*x*_*0*_*-x*_*1*_)^2^+(*y*_*0*_*-y*_*1*_)^2^+(*z*_*0*_*+z*_*1*_)^2^]^1/2^。

The formula for calculating the additional stress generated at any point within a semi-infinite body by a horizontal load *P*_*V*_ is Eqs (4)~(6):

$$\begin{array}{l} \sigma_{x2}=\frac{P_{H}(x_{0}-x_{1})}{8\pi (1-\mu )}\{\frac{-(1-2\mu )}{R_{1}^{3}}-\frac{3{\left(x_{0}-x_{1}\right)}^{2}}{R_{1}^{5}}+\frac{\left(1-2\mu )(5-4\mu \right)}{R_{2}^{3}}-\frac{3(3-4\mu ){\left(x_{0}-x_{1}\right)}^{2}}{R_{2}^{5}}-\\ \;\frac{4(1-\mu )(1-2\mu )}{R_{2}{\left(R_{2}+z_{0}+z_{1}\right)}^{2}}(3-\frac{{\left(x_{0}-x_{1}\right)}^{2}(3R_{2}+z_{0}+z_{1})}{R_{2}^{2}(R_{2}+z_{0}+z_{1})})+\frac{6z_{1}}{R_{2}^{5}}[3z_{1}+(3-2\mu )(z_{0}+z_{1})+\frac{5z_{0}{\left(x_{0}-x_{1}\right)}^{2}}{R_{2}^{2}}]\} \end{array}$$ (5)

$$\begin{array}{l} \sigma_{y2}=\frac{P_{H}(y_{0}-y_{1})}{8\pi (1-\mu )}\{\frac{\left(1-2\mu \right)}{R_{1}^{3}}-\frac{3{\left(y_{0}-y_{1}\right)}^{2}}{R_{1}^{5}}+\frac{\left(1-2\mu )(3-4\mu \right)}{R_{2}^{3}}-\frac{3(3-4\mu ){\left(y_{0}-y_{1}\right)}^{2}}{R_{2}^{5}}-\\ \;\frac{4(1-\mu )(1-2\mu )}{R_{2}{\left(R_{2}+z_{0}+z_{1}\right)}^{2}}(1-\frac{{\left(y_{0}-y_{1}\right)}^{2}(3R_{2}+z_{0}+z_{1})}{R_{2}^{2}(R_{2}+z_{0}+z_{1})})+\frac{6z_{1}}{R_{2}^{5}}[z_{1}-(1-2\mu )(z_{0}+z_{1})+\frac{5z_{0}{\left(y_{0}-y_{1}\right)}^{2}}{R_{2}^{2}}]\} \end{array}$$ (6)

$$\begin{array}{l} \sigma_{z2}=\frac{P_{H}(x_{0}-x_{1})}{8\pi (1-\mu )}\{\frac{\left(1-2\mu \right)}{R_{1}^{3}}-\frac{3{\left(z_{0}-z_{1}\right)}^{2}}{R_{1}^{5}}-\frac{\left(1-2\mu \right)}{R_{2}^{3}}-\frac{3(3-4\mu ){\left(z_{0}+z_{1}\right)}^{2}}{R_{2}^{5}}+\\ \;\frac{6z_{1}}{R_{2}^{5}}[z_{1}+(1-2\mu )(z_{0}+z_{1})+\frac{5z_{0}(z_{0}+z){z_{1}}^{2}}{R_{2}^{2}}]\} \end{array}$$ (7)

In order to express Eqs succinctly in the latter, Eqs (2)~(7) are abbreviated as respectively: _*x1*_ = (*x*_*0*_,*y*_*0*_,*z*_*0*_,*x*_*1*_,*y*_*1*_,*z*_*1*_,*P*_*v*_),*σ*_*y1*_ = (*x*_*0*_,*y*_*0*_,*z*_*0*_,*x*_*1*_,*y*_*1*_,*z*_*1*_,*P*_*v*_), *σ*_*z1*_ = (*x*_*0*_,*y*_*0*_,*z*_*0*_,*x*_*1*_,*y*_*1*_,*z*_*1*_,*P*_*v*_),*σ*_*x2*_ = (*x*_*0*_,*y*_*0*_,*z*_*0*_,*x*_*1*_,*y*_*1*_,*z*_*1*_,*P*_*h*_), *σ*_*y2*_ = (*x*_*0*_,*y*_*0*_,*z*_*0*_,*x*_*1*_,*y*_*1*_,*z*_*1*_,*P*_*h*_), *σ*_*z1*_ = (*x*_*0*_,*y*_*0*_,*z*_*0*_,*x*_*1*_,*y*_*1*_,*z*_*1*_,*P*_*h*_).

### Elastic foundation beam theory

**Fig 2:**
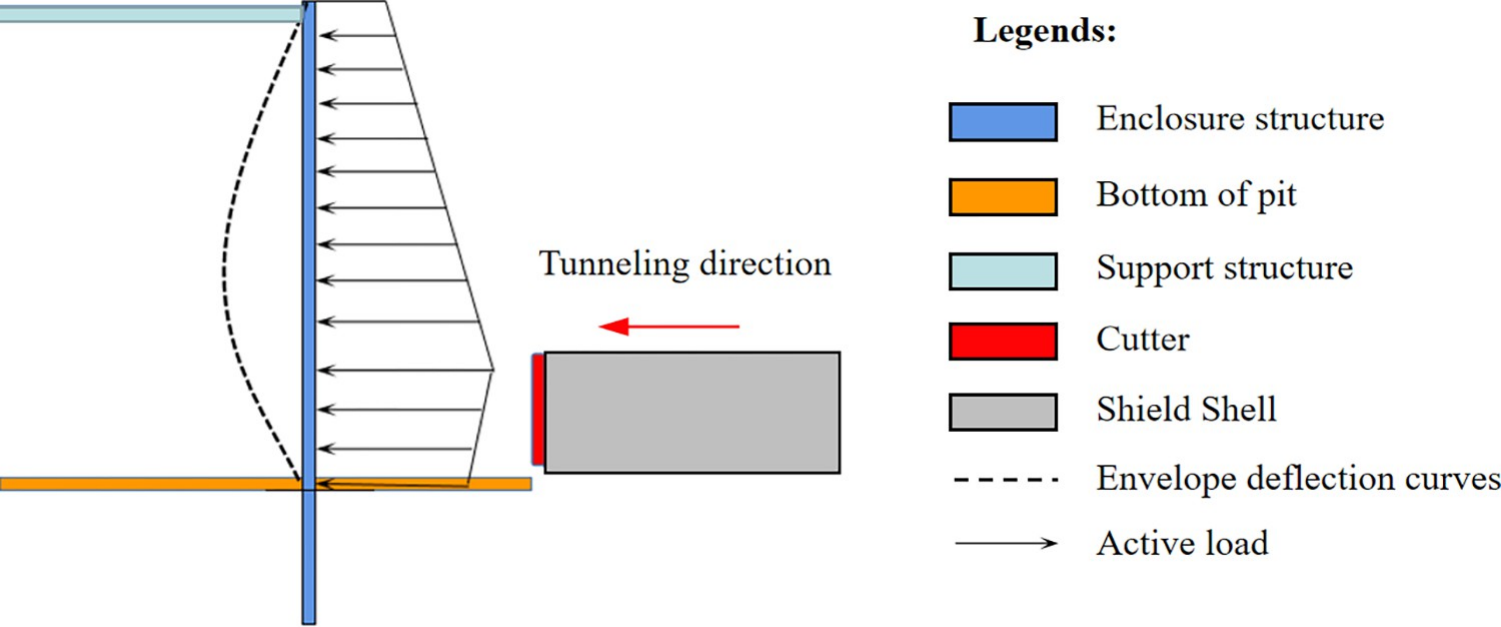
Foundation beam and simplified model.

The vertical foundation beam method is commonly employed to calculate the deformation of enclosure structures. In this study, the conventional elastic foundation beam is simplified further. In a vertical foundation beam, the enclosure structure’s two ends undergo rotation and deformation due to active and passive earth pressure. However, it is clear that treating the enclosure structure as a vertical elastic foundation beam cannot consider the support effect of the first layer of support structure on the enclosure structure. Consequently, it is not suitable for calculating the additional effects resulting from shield tunneling construction factors. Therefore, this study modifies the vertical elastic foundation beam approach by disregarding the rotation and displacement at the bottom position of the enclosure structure, as well as the displacement at the top. Instead, the enclosure structure is treated as a beam with fixed supports at both ends. Fig 2 provides a visual comparison between the vertical foundation beam and the simplified model utilized in this study.

**Fig 3:**
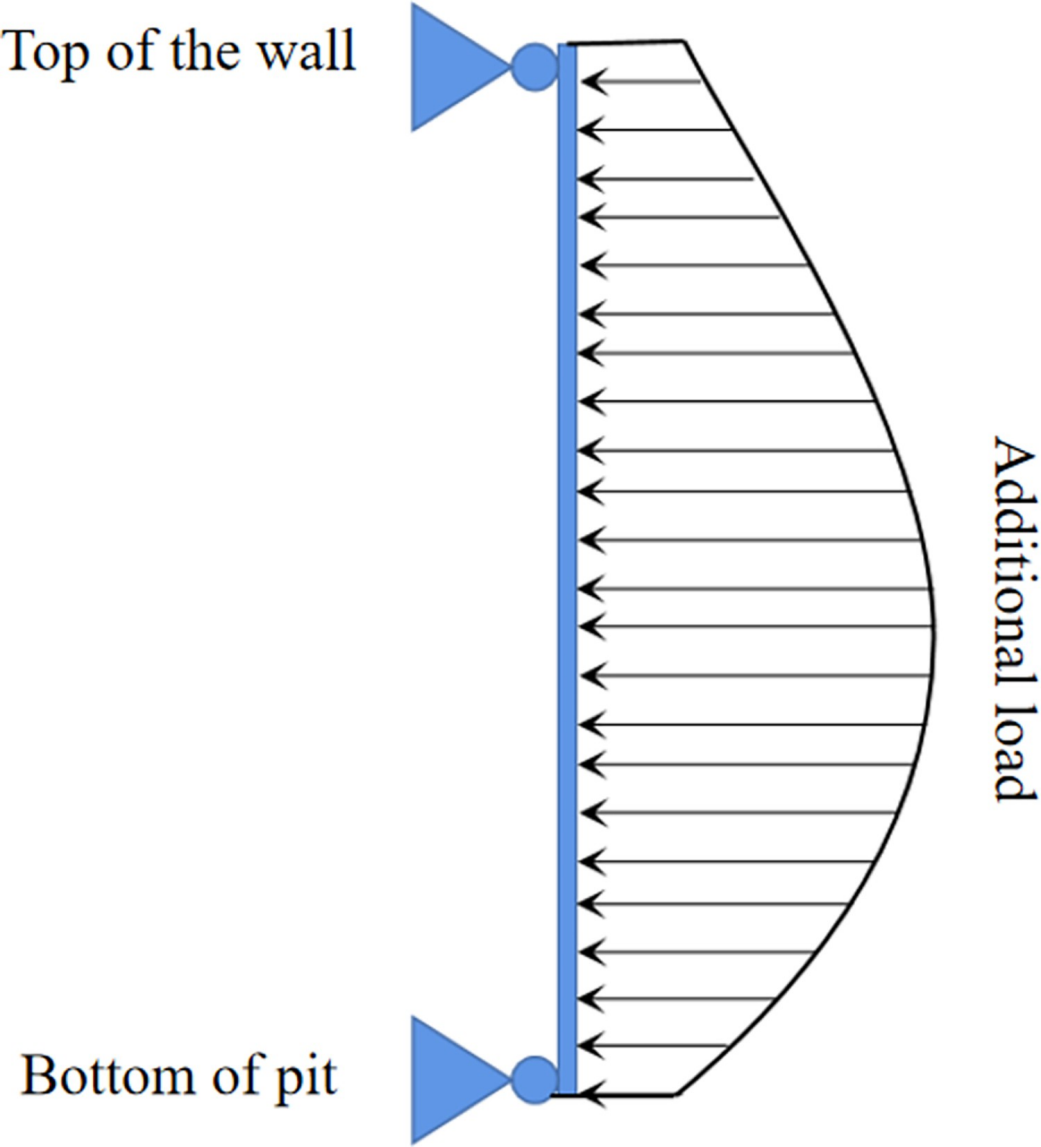
Simplified model diagram.

The simplified model used in this study is shown in Fig 3.

The enclosure structure, spanning from the bottom to the top of the wall, is simplified as a vertically oriented beam and discretized into *n* elements. Each element undergoes horizontal movement and horizontal rotation under the influence of the additional load. The displacement and external load of each element satisfy the following requirements based on the stiffness matrix formula of structural mechanics:

$$\left[\begin{array}{c} F_{M}^{e}\\ F_{s}^{e}\\ M_{i}^{e}\\ F_{\Delta }^{e}\\ F_{S}^{e}\\ M_{j}^{e} \end{array}]=[\begin{array}{cccccc} \frac{EA}{l} & 0 & 0 & -\frac{EA}{l} & 0 & 0\\ 0 & \frac{12EI}{l^{3}} & \frac{6EI}{l^{2}} & 0 & -\frac{12EI}{l^{3}} & -\frac{6EI}{l^{2}}\\ 0 & \frac{6EI}{l^{2}} & \frac{4EI}{l} & 0 & -\frac{6EI}{l^{2}} & \frac{2EI}{l}\\ -\frac{EA}{l} & 0 & 0 & \frac{EA}{l} & 0 & 0\\ 0 & -\frac{12EI}{l^{3}} & -\frac{6EI}{l^{2}} & 0 & \frac{12EI}{l^{3}} & -\frac{6EI}{l^{2}}\\ 0 & \frac{6EI}{l^{2}} & \frac{2EI}{l} & 0 & -\frac{6EI}{l^{2}} & \frac{4EI}{l} \end{array}]=[\begin{array}{c} u_{i}^{e}\\ v_{i}^{e}\\ \varphi_{i}^{e}\\ u_{j}^{e}\\ v_{j}^{e}\\ \varphi_{j}^{e} \end{array}\right]$$ (8)

In the Eq (8), *F*_*M*_^*e*^ is the magnitude of axial force applied to the *i*-th unit, which will cause compression deformation *u*_*i*_^*e*^ of the unit. *F*_*Si*_^*e*^ is the shear force of the *i*-th unit, which will cause the horizontal displacement *v*_*i*_^*e*^ of the unit. *M*_*i*_^*e*^ is the magnitude of the horizontal bending moment on the *i*-th unit, which will cause horizontal deflection *φ*_*i*_^*e*^ of the unit. *F*_*Δ*_^*e*^ is the magnitude of axial force applied to the *j*-th unit, which will cause compression deformation *u*_*j*_^*e*^ of the unit. *F*_*Sj*_^*e*^ is the shear force of the *j*-th unit, which will cause the horizontal displacement *v*_*j*_^*e*^ of the unit. *M*_*j*_^*e*^ is the magnitude of the horizontal bending moment on the *j*-th unit, which will cause horizontal deflection *φ*_*j*_^*e*^. The *i*-th unit and *j*-th unit are adjacent units. *E* is the elastic modulus of the structure. *A* is the cross-sectional area of the structure. *L* is the length of the unit. *I* is the moment of inertia of the structure.

## Calculation of additional load and deformation

### Additional loads

#### Cutterhead additional thrust

**Fig 4:**
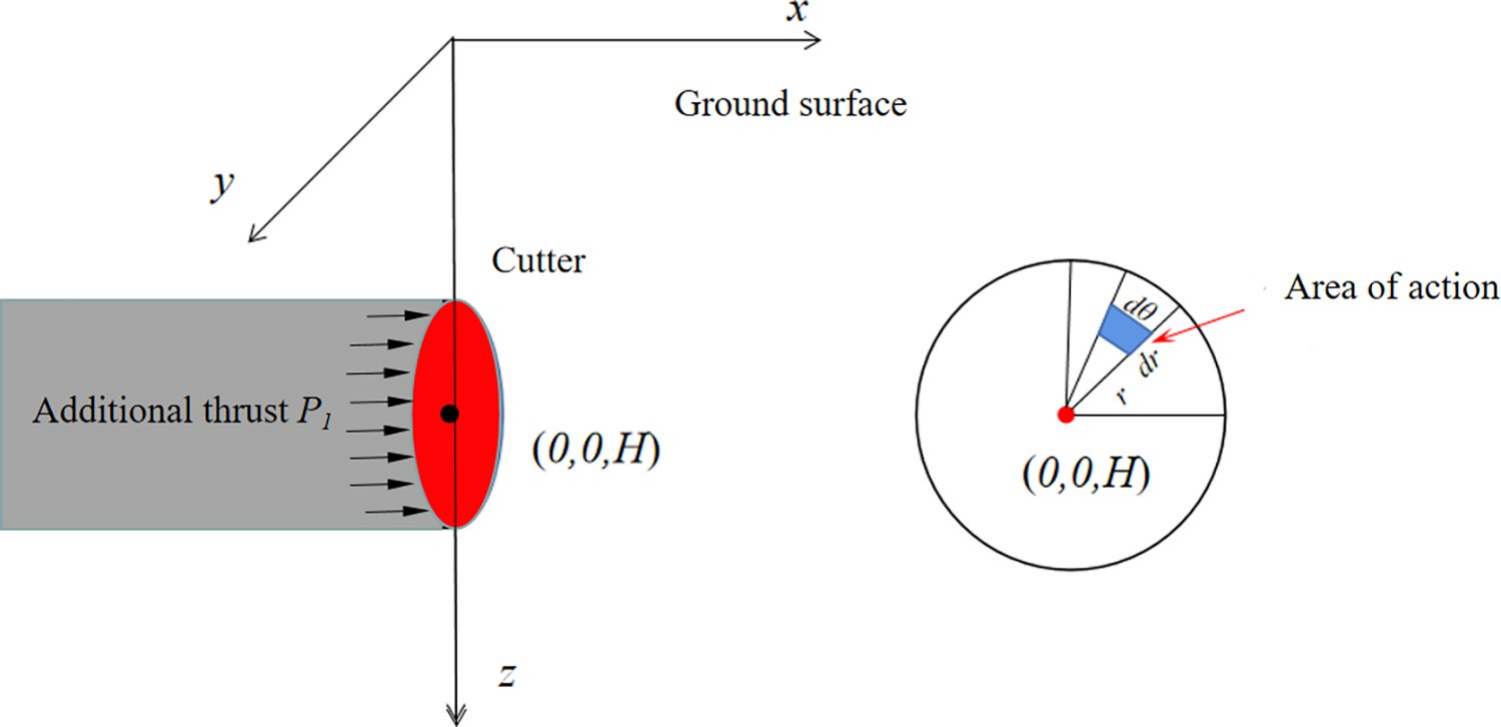
Calculation model for additional thrust.

The center point of the cutterhead is located at coordinates (0, 0, *H*), and the shield machine cutterhead has a diameter of *D*. Assuming a uniform distribution of thrust on the cutterhead, the action area is represented by *dθdr*. If the distance between the action position and the center point of the cutterhead is denoted as *r*, the integration area for the additional thrust covers the entire surface of the cutterhead, with an integration angle range of [-π, π] and an integration length range of [0, *D/2*], as depicted in Fig 4. By substituting the integration region into Eqs (2) to (4), the formula for calculating the additional thrust caused by the cutterhead’s additional thrust in the surrounding environment is obtained as shown in Eq (9).


$$\sigma_{x2p}=\int_{0}^{R}\int_{-\pi }^{\pi }(x_{0},y_{0}-\cos\theta ,z_{0}-H+r\sin\theta ,x_{1},y_{1},z_{1},p)drd\theta$$ (9)

#### Force of friction between shield and soil

**Fig 5:**
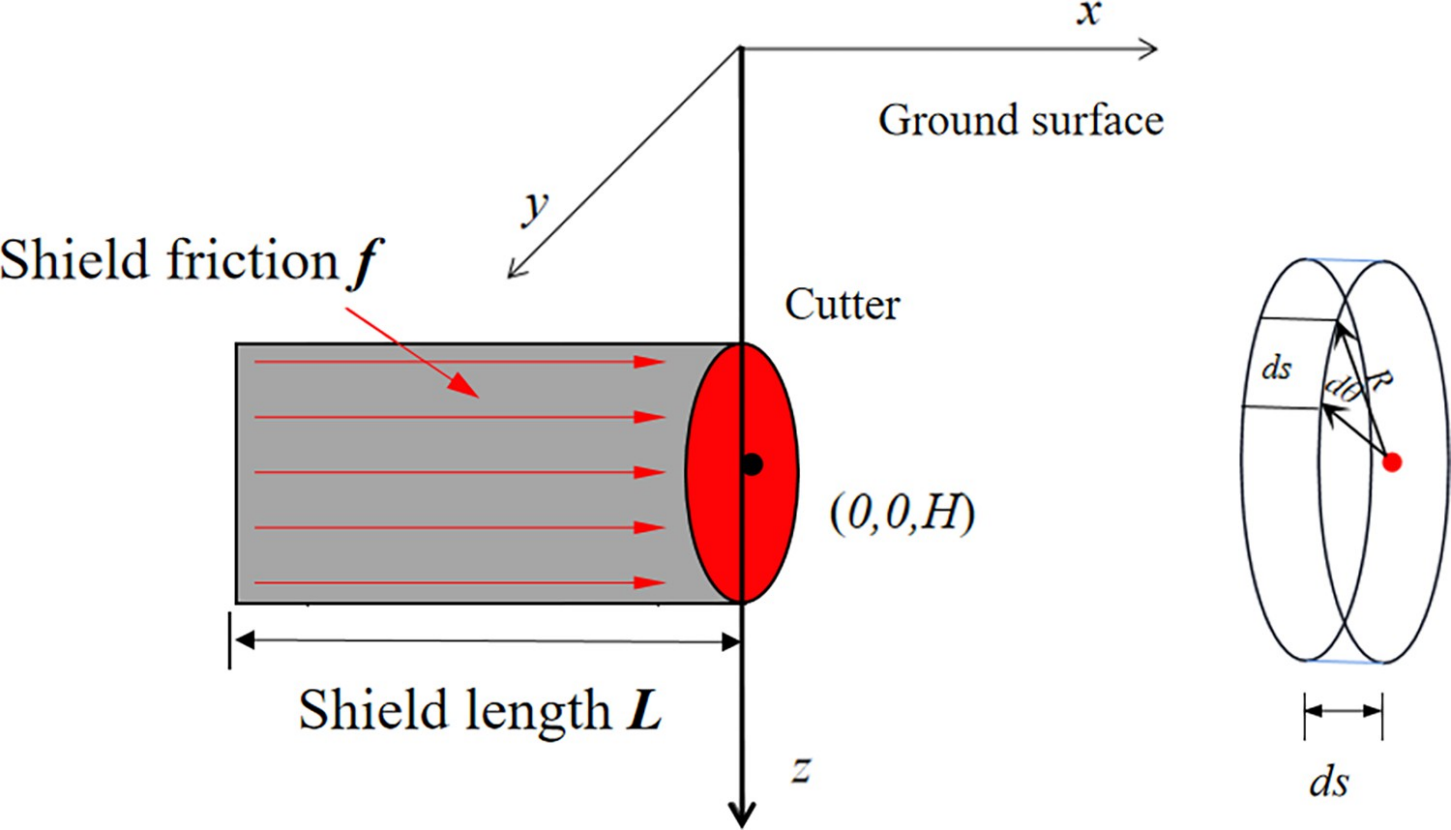
Calculation model for friction force.

Assuming a uniformly distributed frictional resistance between the shield shell and the surrounding soil within the shield scope, the frictional resistance acts in the positive direction along the *y*-axis. The unit area of frictional resistance on the shield shell is represented by *Rdsdθ*. When considering the effect of frictional resistance on the shield shell, the integration area encompasses the entire surface of the shield shell. The integration angle range is [-π, π], and the integration length range is [0, *L*], as illustrated in Fig 5. By substituting the integration region into Eqs (2) to (4), the formula for calculating the additional thrust caused by the frictional resistance between the shield shell and the surrounding soil is obtained as shown in Eq (10).


$$\sigma_{x2f}=\int_{0}^{L}\int_{-\pi }^{\pi }(x_{0}-s,R\cos\theta ,z_{0}-H+R\sin\theta ,x_{1},y_{1},z_{1},f)dsd\theta$$ (10)

#### Additional grouting pressure

**Fig 6:**
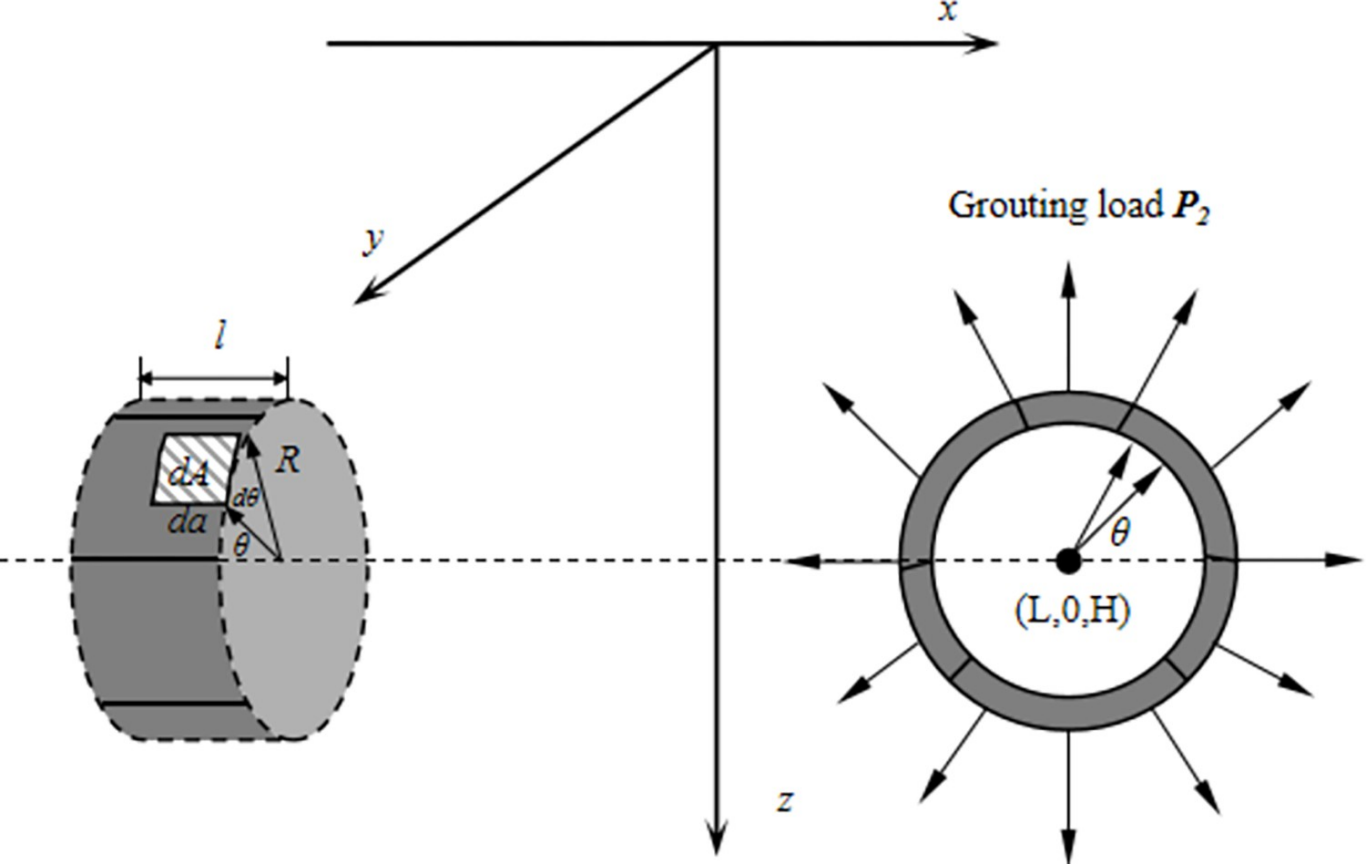
Calculation model for additional grouting pressure.

Assuming a uniform distribution of grouting pressure along the shield shell, the grouting pressure acts within the length of a single ring segment behind the shield tail. The integral interval for calculating the additional stress caused by the grouting pressure is [-π, π], and the integral length is equal to the length *l* of the single ring segment, as illustrated in Fig 6. To consider the effect of the additional grouting pressure, it is necessary to decompose it into two components: horizontal and vertical pressure. The horizontal component, *qRcosθdθ*, acts along the y-axis. Therefore, a coordinate transformation is required for Eqs (1) to (3). The variables ξ and ξ_0_ in the Eqs (2)~(7) need to be transformed into *η* and *η*_*0*_. The formula for calculating the additional stress, obtained after the transformation, is represented by Eqs (11), (12).


$$\sigma_{x2q}=\int_{L}^{L+l}\int_{-\pi }^{\pi }(x_{0}-L-a,y_{0}-R\cos\theta ,z_{0}-H+R\sin\theta ,x_{1},y_{1},z_{1},q)dad\theta$$ (11)

$$\sigma_{x1q}=\int_{L}^{L+l}\int_{-\pi }^{\pi }(x_{0}-L-a,y_{0}-R\cos\theta ,z_{0}-H+R\sin\theta ,x_{1},y_{1},z_{1},q)dad\theta$$ (12)

In conclusion, Eqs (9) to (11) can be utilized to compute the additional stress in the surrounding strata resulting from the bulkhead additional thrust, frictional force, and additional grouting pressure during shield tunneling. The additional stress at any given point can be determined using the following Eq (13):

$$\sigma_{x}=\sigma_{x2p}+\sigma_{x2f}+\sigma_{x2q}+\sigma_{x1q}$$ (13)

### Deformation calculation

Substitute the calculated additional load caused by shield tunneling into Eq (8) and make the additional load equal to *F*_*M*_^*e*^, so that the horizontal deformation *v*_*i*_^*e*^ of the enclosure structure can be obtained.

## Engineering example verification

### Engineering background

**Fig 7:**
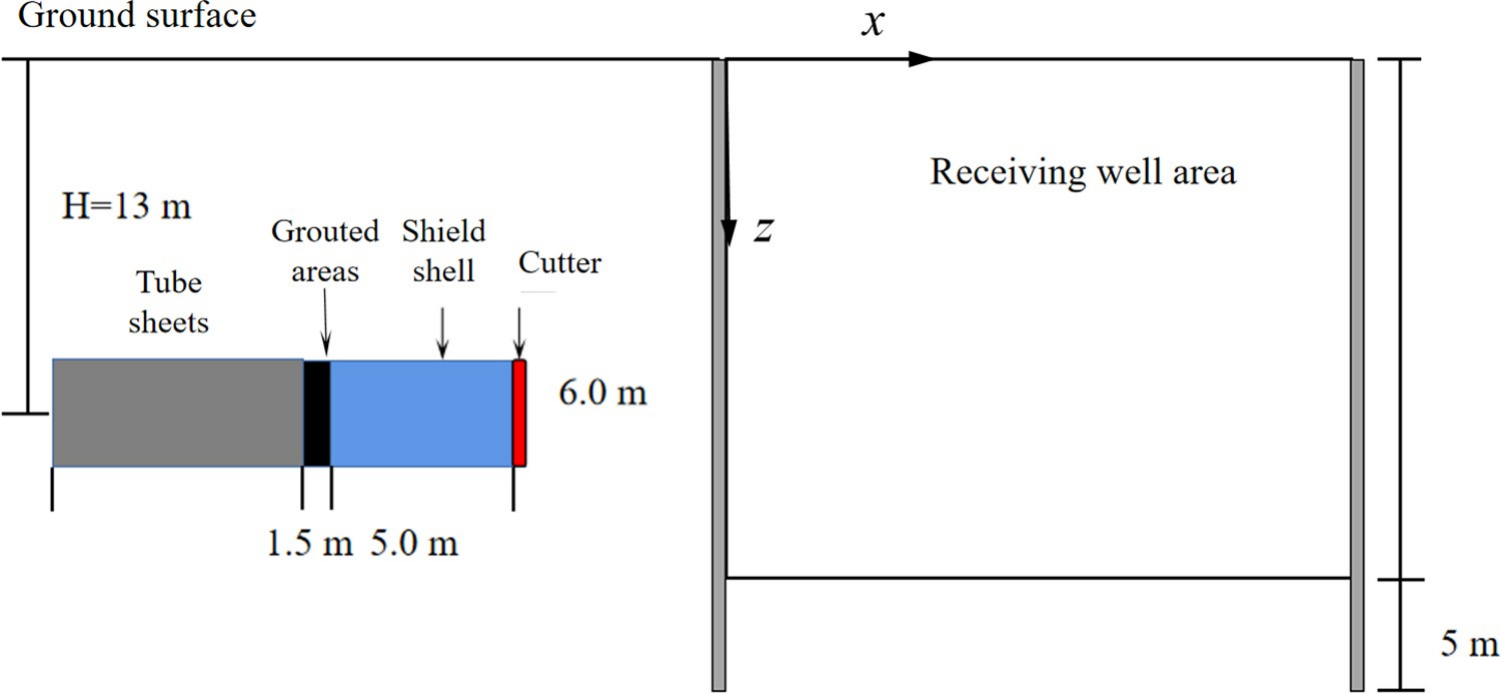
Location diagram.

This study focuses on a specific subway engineering project constructed using shield tunneling as the context. The tunnel arch is buried at a depth of 13.4–15.2m, with an inner diameter of the shield tunnel segment measuring 5.5m. The segment thickness is 350mm, while the outer diameter of the segment is 6.2m, and the segment width is 1.5m. The receiving well enclosure structure has dimensions of 10m×20m. The enclosure structure is supported by a C30 cast-in-place diaphragm wall, and the first layer of support consists of a concrete support, ensuring a secure connection without any relative movement between the concrete support and the enclosure structure. The size and location of the tunnel and the excavated well are depicted in Fig 7.

### Numerical simulation model

A numerical simulation model is established using Midas GTS. The calculation parameters are applied to the numerical simulation model to obtain the solutions, which are compared with theoretical analysis and analyzed for the applicability of the theoretical formulas.

#### Model size and boundary conditions

The numerical simulation model mainly includes geological structure, shield tunnel structure, and receiving well enclosure structure. The shield tunnel structure includes tunnel segments and tunnel excavation parts. The enclosure structure of the receiving well includes the excavation soil inside the receiving well and its external wall structure. The main analysis object of this study is the external wall of the receiving well. The size of the receiving well in a single line tunnel is 20 m×10 m×25 m. The outer diameter of the shield machine is 6.1m and the length is 5 m.

**Fig 8:**
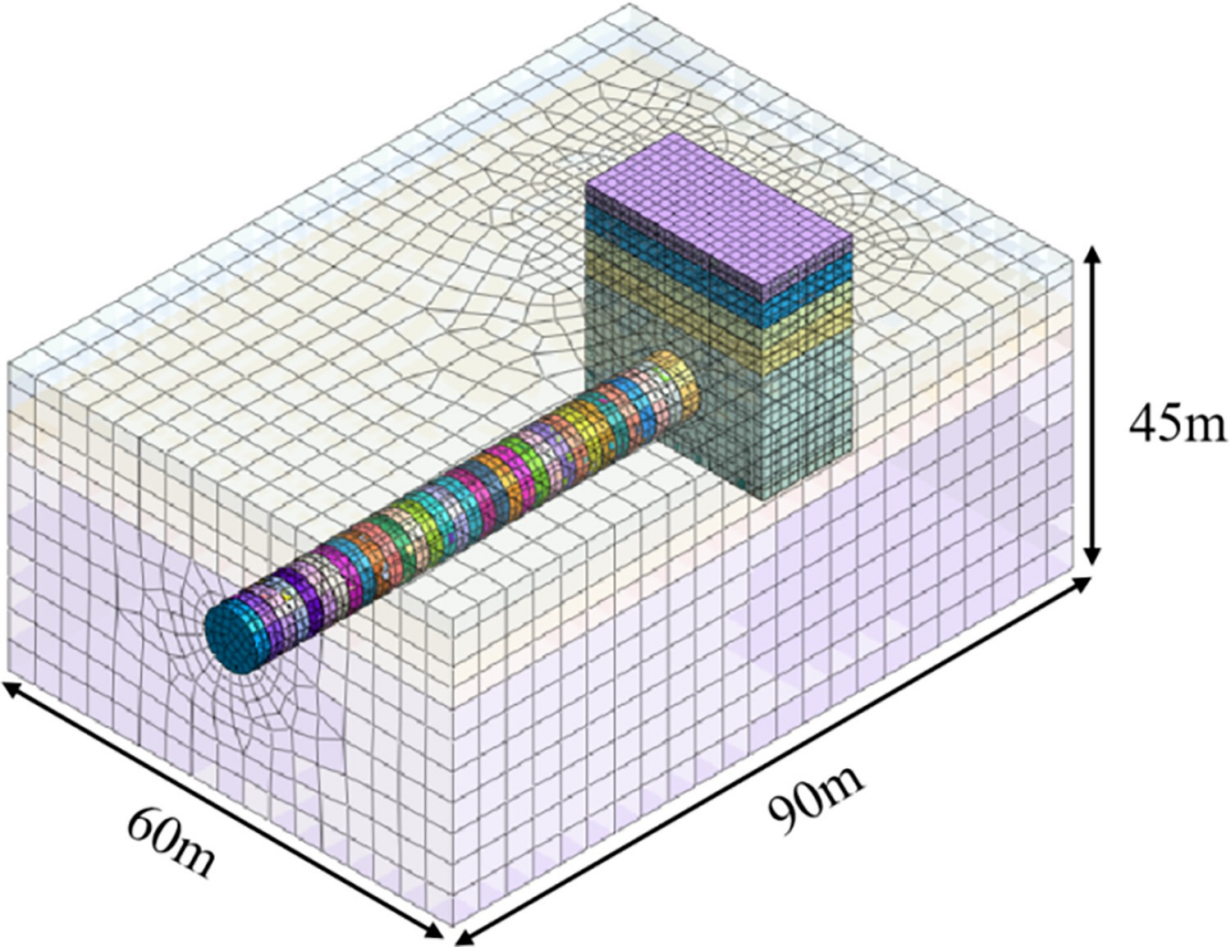
Numerical simulation model.

The boundary conditions applied to the model are as follows: fixed constraints are set at the bottom of the model to restrict horizontal and vertical displacements of the bottom. The top of the model is designated as a free surface, disregarding any influences beyond the model’s boundaries, and only limiting normal displacements of the model’s sides [22–24]. The numerical simulation model is illustrated in Fig 8.

#### Material parameters

In the model, the soil parameters adopt the Mohr-Coulomb constitutive model [25], and the soil’s mechanical parameters were mainly obtained by field and laboratory tests. The cutter head, and shield shell parameters were mainly obtained from the design drawings. The longitudinal and circumferential joints of the segments were not considered in the model. The shield tunnel joints’ influence on the stiffness of the tunnel can be considered through stiffness reduction. According to the equivalent continuity model and stiffness calculation equation of the shield tunnel proposed by Zhou, et al. [26], the reduction factor of the shield tunnel segment was calculated as 0.88. The segments were made of C30 grade concrete, and the elastic modulus E, Poisson’s ratio, and gravity were 25.9 GPa, 0.2, and 25 kN/m^3^, respectively. After the stiffness was reduced, the elastic modulus of the segment was 22.8 GPa. Notably, owing to the presence of the cutter-head and the chamber, the shell shield elements had a stiffness that was 100 times higher than the elastic modulus of the segment, and the deformations were ignored. Because the grout material’s elastic modulus is time-dependent, an initial elastic modulus of 40 MPa was assigned to the unsolidified area of the grout to simulate the mechanical properties of fresh grout.

The soil strength parameters and construction parameters used in the theoretical calculation formula represent average values within the region. The specific calculation parameters can be found in Table 1.

**Table 1:** Calculation parameters.

*D/*(m)	*H/*(m)	*γ/*(kN·m^3^)	*L/*(m)	*l/*(m)	*μ/*(m)
6.0	13.0	18.1	5.0	1.5	0.28
*p/*(kPa)	*f/*(kPa)	*q/*(kPa)	E(GPa)		
15.0	20.0	10.0	22.3		

#### Excavation steps

The model calculation process can be divided into three main steps:

Step 1: Assign soil layer parameters and apply gravity to achieve the initial stress equilibrium state of the model;

Step 2: Reset the displacement field in step 1 to zero and retain the initial stress field. Assign corresponding materials to the surrounding structure of the receiving well, and use the Beam element of the software to achieve the support effect inside the receiving well. Then, perform calculations to obtain the stress and deformation of the receiving well.

Step 3: Clear the displacement field in Step 2, remove the soil in the excavation area of the shield tunnel, assign materials to the segments, shield shell, cutter head, and apply excavation loads to obtain the final calculation result.

### Analysis of calculation results

#### Deformation in the x-direction of the receiving well

**Fig 9:**
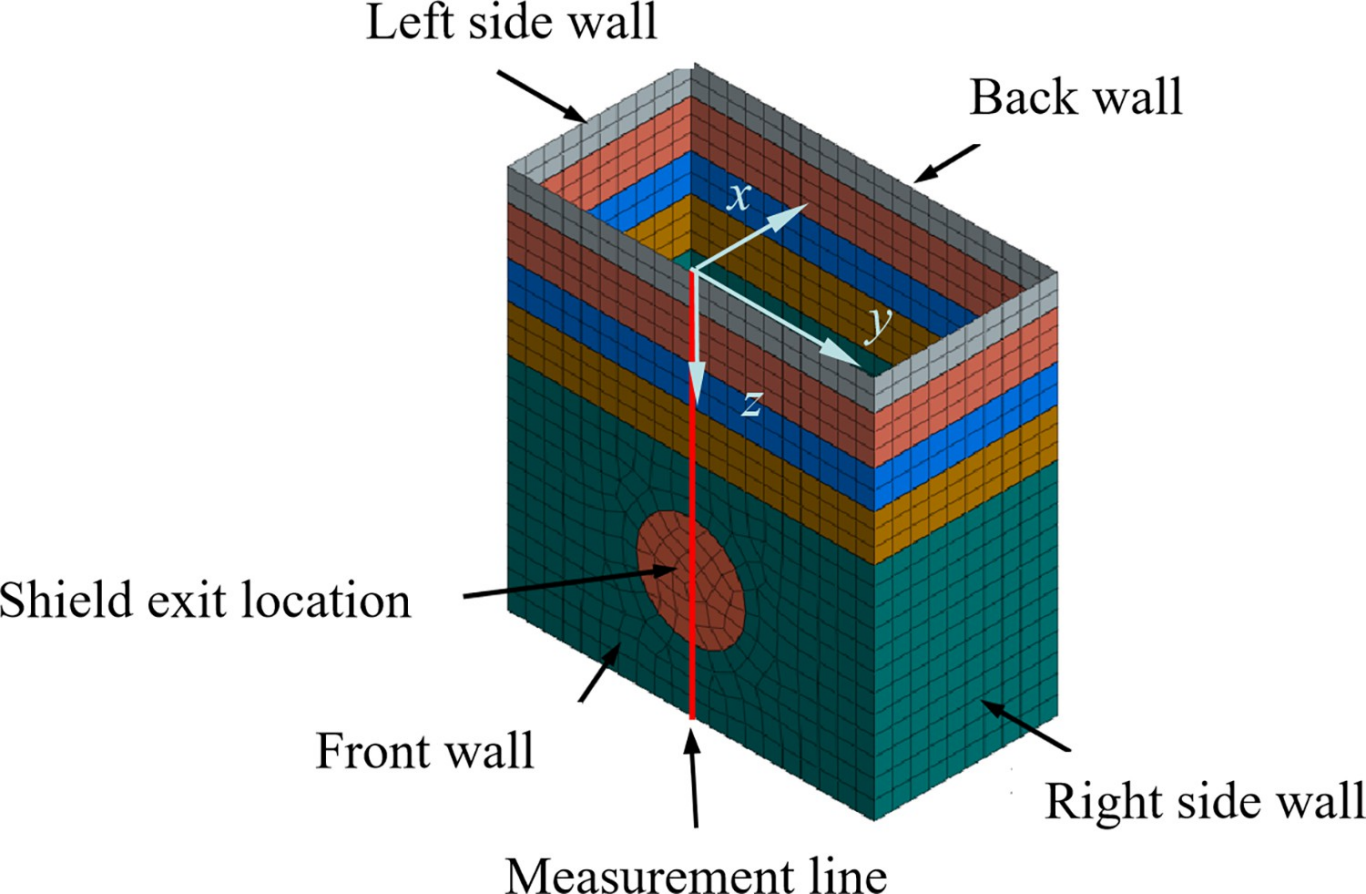
Receiving well model.

The receiving well model is shown in Fig 9. During shield tunneling, the axis position on the external wall of the receiving well experiences the most significant disturbance. Consequently, the deformation in the *x*-direction at the axis position on the external wall of the receiving well is analyzed to validate the accuracy of the numerical simulation model and theoretical calculations.

**Fig 10:**
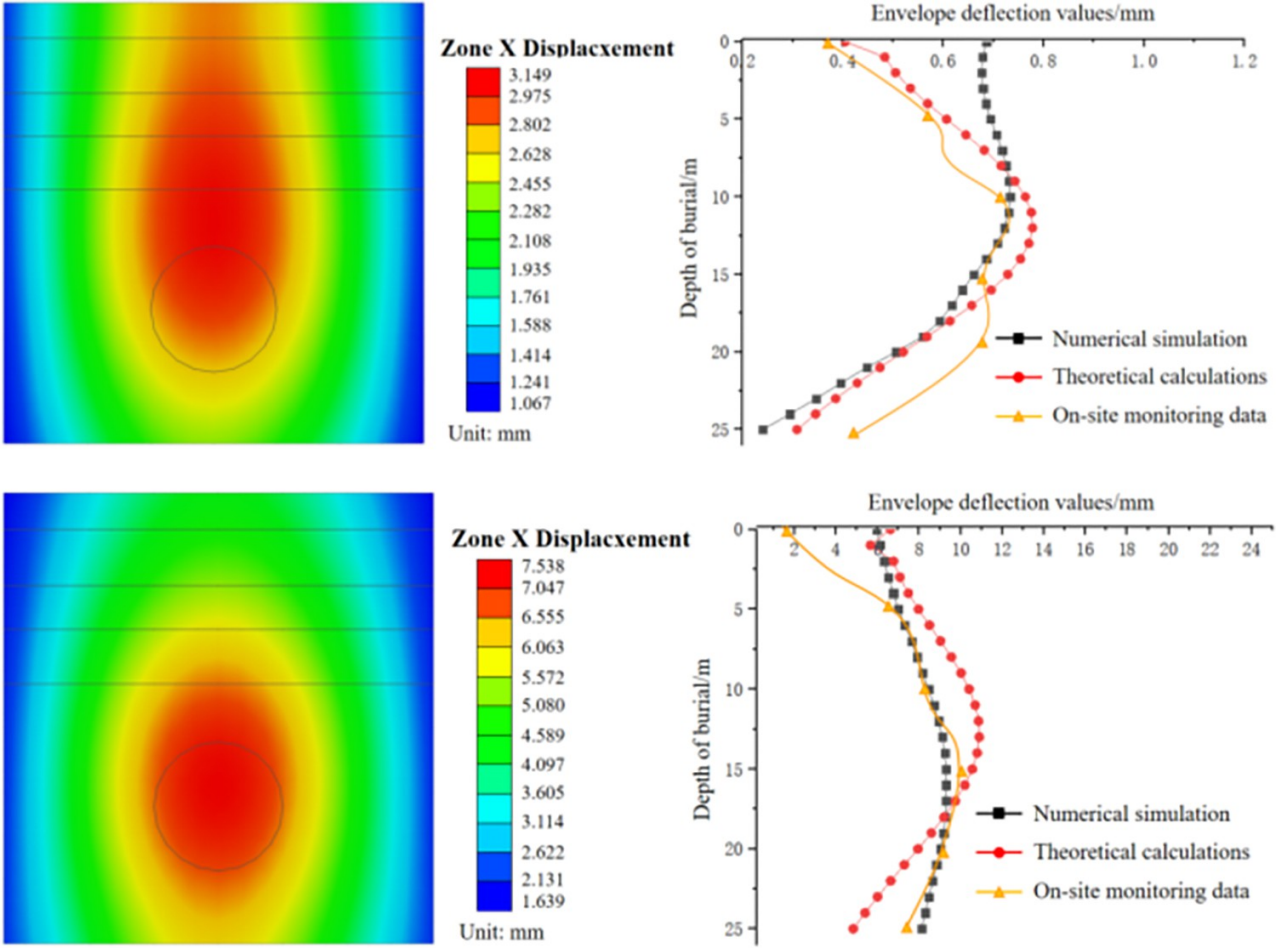
Cloud chart and curve of external wall deformation of receiving well. (a) Shield machine is 20m away from the receiving well. (b) Shield machine is 10 m away from the receiving well.

From Fig 10, it can be obtained that:

1. As the excavation distance of the shield machine increases, the maximum deformation position of the receiving well enclosure structure gradually shifts downwards towards the point where the shield machine exits the tunnel. When the tunnel face is 20 m away from the receiving well wall, the concentrated deformation occurs near the center of the shield machine. At a distance of 10m, the concentrated deformation extends to cover the exit, resulting in a maximum deformation of 6.5 mm.
2. In comparing the results obtained from numerical simulations, theoretical calculations, and on-site deformation monitoring, we have observed a general alignment in the deformation trends of the receiving well as measured by these three methods. However, noteworthy disparities emerge between the theoretical calculation outcomes and the numerical simulation results, particularly when examining excavation at close distances. These disparities can be attributed to two primary factors.

Firstly, the theoretical calculation simplifies the enclosure structure as a beam element. This simplification may not adequately capture the structural deformation characteristics, as it overlooks the plate-like behavior inherent in the structure. Employing plate elements in lieu of beam elements within the theoretical calculation can yield a more accurate representation of the actual deformation state, consequently enhancing the precision of the calculated results.

Secondly, the theoretical calculation fails to account for the formation of plastic zones within the soil during excavation and its consequential impact on soil strength. The analysis exclusively relies on the elastic calculation of a specific cross-section, overlooking the influence of plastic zones. This omission can introduce inaccuracies into the calculated results, as plastic zones exert a significant influence on the soil’s strength and behavior during tunneling.

#### Additional stress

**Fig 11:**
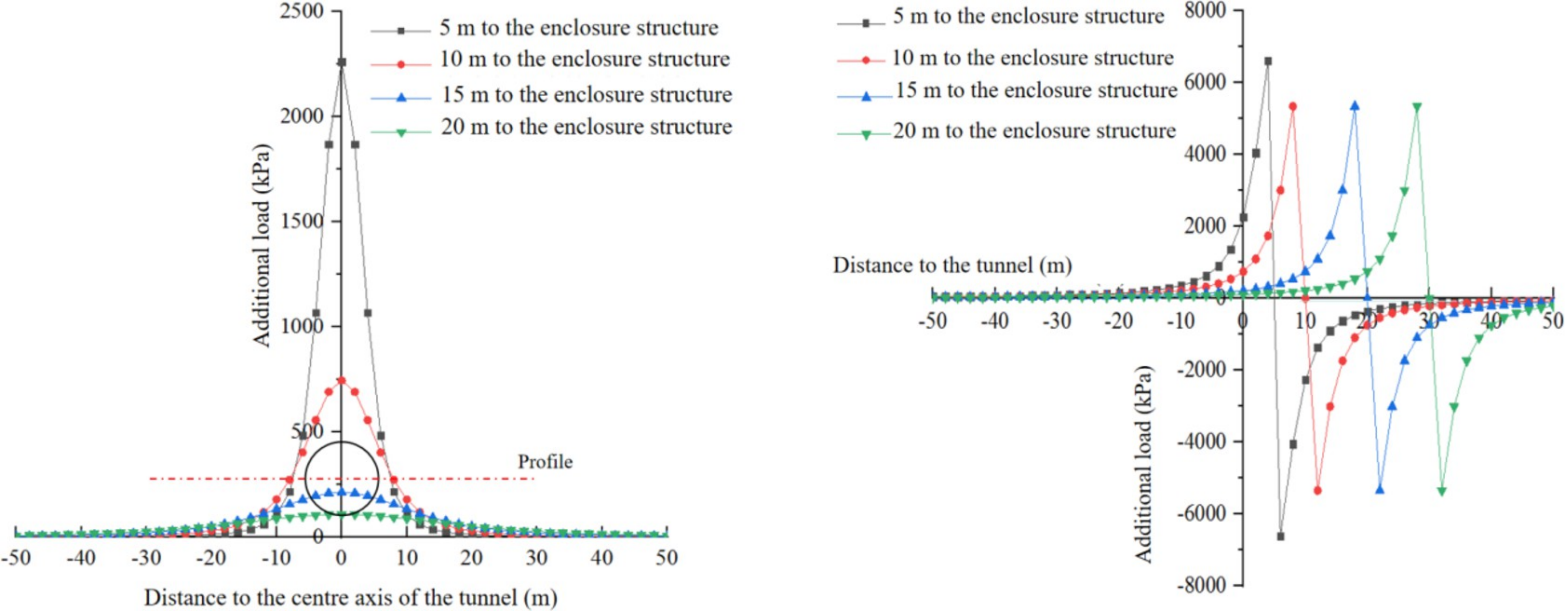
Distribution of additional loads caused by additional thrust. (a) y-direction. (b) x-direction.

**Fig 12:**
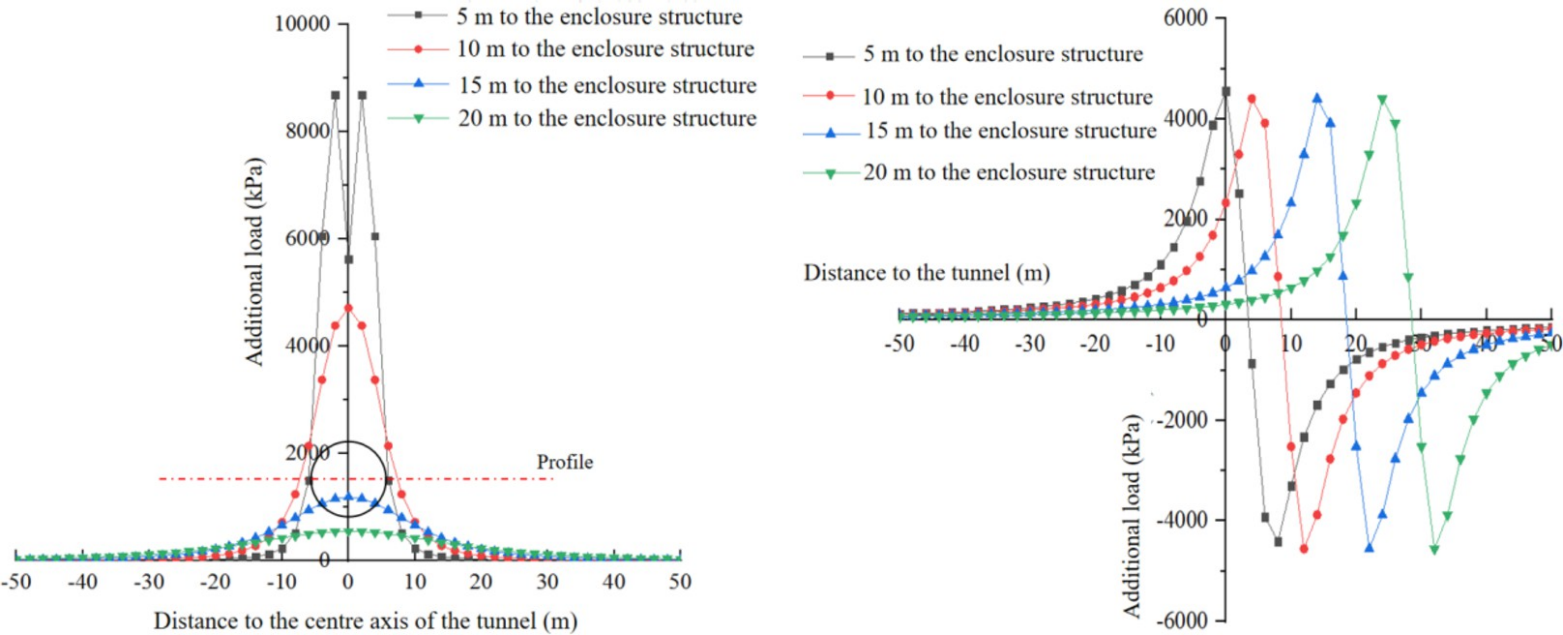
Additional load caused by force of friction between shield and soil. (a) y direction. (b) x direction.

In this study, since monitoring the additional stress in the receiving well during numerical simulations and on-site construction is not feasible, the distribution of additional stress in the receiving well caused by shield tunneling is analyzed using theoretical calculation methods. The additional stress resulting from various construction factors are calculated at a depth of H = 13m in both the transverse and longitudinal directions, considering different distances between the shield machine cutterhead and the side wall of the receiving well (5m, 10m, 20m, and 30m). The calculated results for the additional stress loads are presented in Figs 10 to 12.

In Fig 11, the distribution of additional load caused by the additional thrust in the *y*-direction is presented. On the cross-section of the shield machine, as the distance from the tunnel axis increases, the additional load in the *x*-direction resulting from the additional thrust decreases. The region within 10m on both sides of the tunnel axis experiences higher additional load compared to the regions beyond this range, where the additional load significantly decreases. Moreover, as the distance between the tunnel face and the enclosure structure decreases, the additional load and its increase amplitude in the *x*-direction also increase. For instance, when the distance between the tunnel face and the enclosure structure is 10m, the maximum additional load in the *x*-direction caused by the additional thrust is 745 kPa. However, when the distance is reduced to 5m, the maximum additional load in the *x*-direction reaches 2263 kPa, representing an increase of approximately 200%. Thus, as the distance between the tunnel face and the enclosure structure decreases, the additional load induced by the additional thrust on the enclosure structure becomes larger.

In Fig 11(B), the distribution of additional load caused by the additional thrust in the *x*-direction is illustrated. The additional load in the *x*-direction exhibits an antisymmetric distribution, with the center of symmetry located at the cutterhead. This implies that the additional thrust does not generate additional load in the *x*-direction on the soil layer within the same plane where the cutterhead is situated. When the cutterhead of the shield machine is at different positions, the maximum additional load in the *x*-direction is basically the same, with a maximum load of 3010 kPa, 2.5m ahead and 2.5m behind the cutterhead. The additional thrust induces the soil ahead of the cutterhead to generate additional load in the positive direction of the *x*-axis, while causing the soil behind the cutterhead to generate additional load in the opposite direction of the *x*-axis.

Fig 12 depicts the additional load in the *x*-direction caused by the force of friction between the shield and soil. The distribution range of the additional load in the *x*-direction due to the force of friction is broader compared to the additional thrust. However, the distribution pattern is basically consistent with the distribution pattern of the additional load in the *x*-direction caused by the bulkhead additional thrust. The additional load exhibits axial symmetry in the *y*-direction, with a symmetry axis at y = 0. The additional load is antisymmetric in the *x-*direction with the center of symmetry at the centerline of the shield shell. When the calculation point is located above the centerline of the shield shell, the half of the shield shell ahead of the centerline generates additional load in the *x*-negative direction, while the other half produces additional load in the *x*-positive direction at the calculation point. The superposition of these loads results in an additional load in the *x*-direction of 0.

The additional load in the *x*-direction caused by the force of friction between the shield and soil is significantly greater than that caused by the bulkhead additional thrust. This discrepancy arises because the integral range of the bulkhead additional thrust is much smaller compared to the integral range of the force of friction between the shield and soil.

In the *y*-direction, the additional load caused by the bulkhead additional thrust undergoes a mutation at y = 0 when the tunnel face is 5 m away from the enclosure structure. The reason for the mutation is that as the distance decreases, the size effect of the cutterhead on the enclosure structure becomes greater. Due to the symmetrical distribution of frictional resistance on both sides of the shield shell, the superimposed effect on the center point will weaken the additional load. However, when the distance between the tunnel face and the enclosure structure is far, the symmetrical effect of frictional resistance on both sides is smaller than that of the distance and the length of the enclosure structure. As the distance shortens, the size effect of frictional resistance on the distance and the length of the enclosure structure gradually increases. When the distance reaches a certain range, the additional load in the middle range will be less than that on both sides.

**Fig 13:**
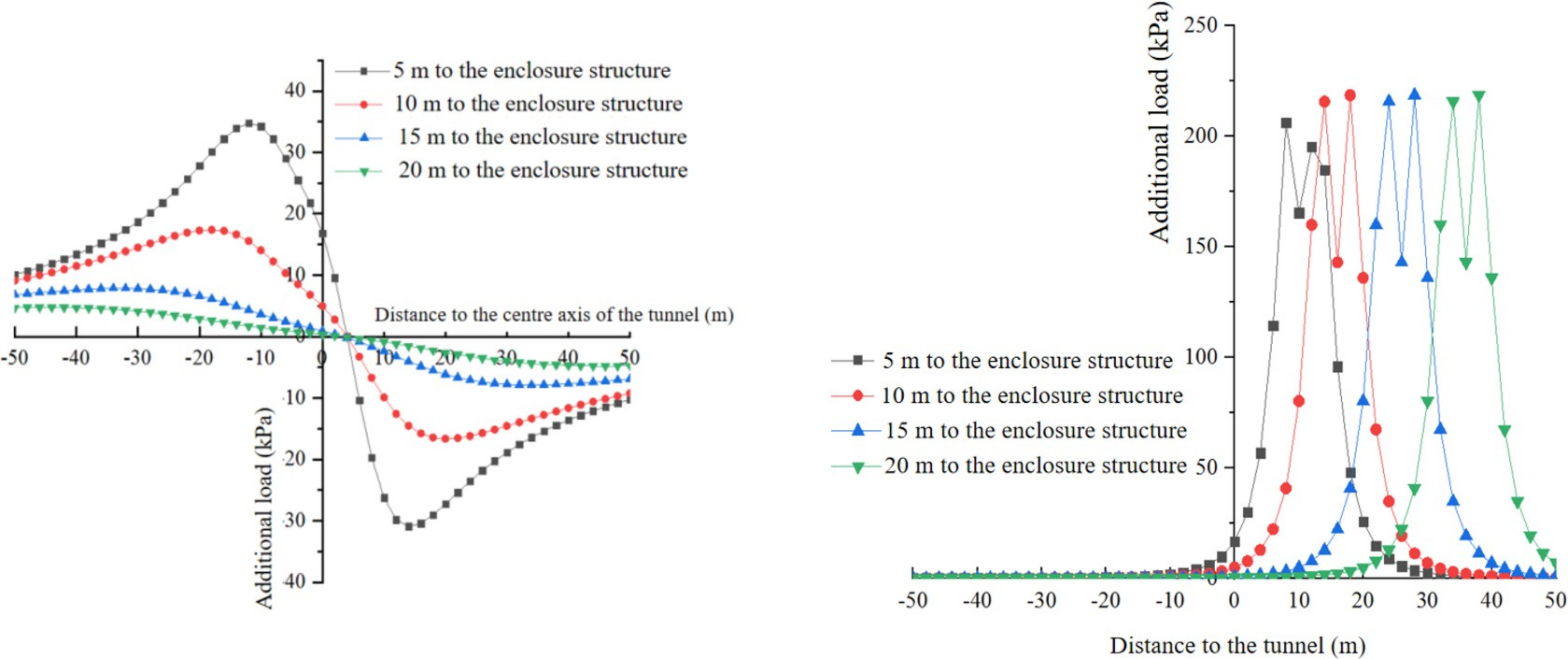
Additional load caused by additional grouting pressure. (a) y direction. (b) x direction.

Fig 13 presents the distribution of additional load in the x-direction resulting from the additional grouting pressure. Fig 13(A) shows that, the additional load in the *x*-direction is distributed asymmetrically in the y-direction, which differs from that caused by bulkhead additional thrust and the force of friction between shield and soil. The maximum value of the additional load occurs approximately at the midpoint of the grouting position. As the distance from the grouting position increases, the additional load gradually decreases. The additional load in the *x-*direction caused by the additional grouting pressure has little influence on the tunnel face, and the impact range is roughly within the range of the three ring segments near the grouting position. The maximum additional load is only 200 kPa, which can be disregarded when compared to the bulkhead additional thrust and the force of friction between the shield and soil.

**Fig 14:**
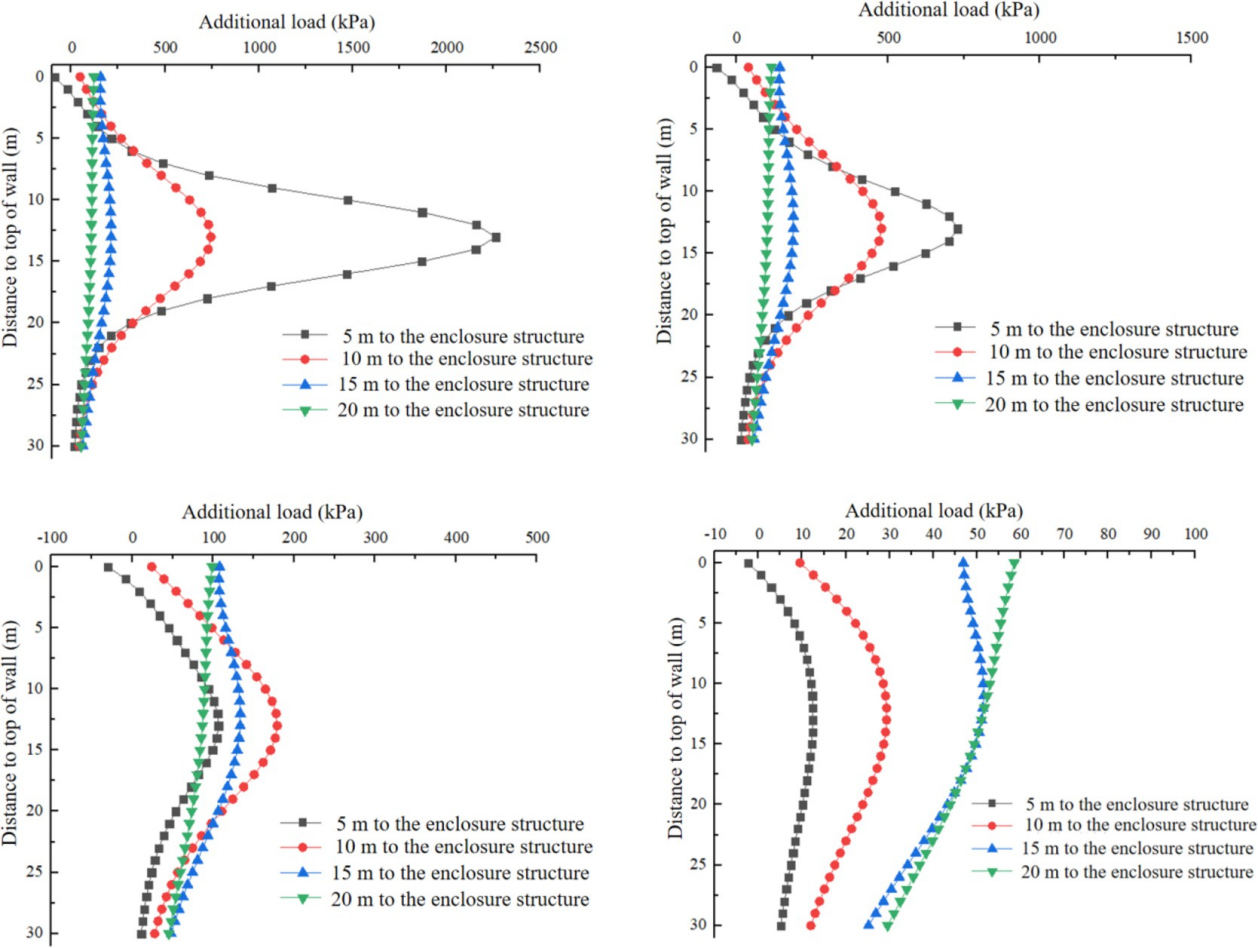
Additional load caused by additional thrust. (a) y = 0 m. (b) y = 10 m. (c) y = 20 m. (d) y = 30 m.

**Fig 15:**
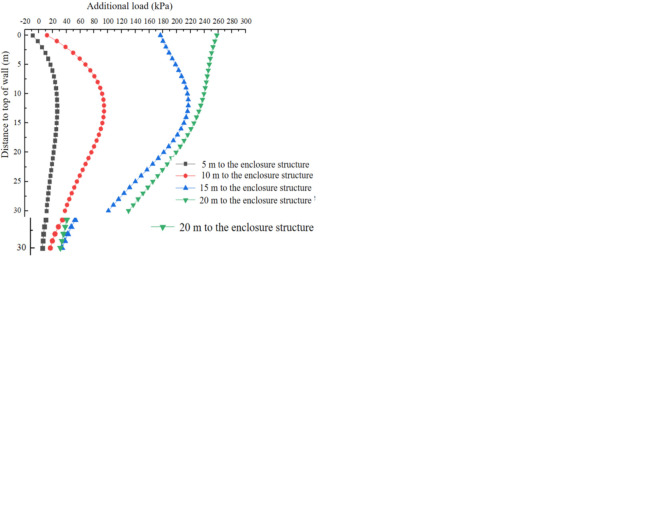
Additional load caused by frictional resistance of shield shell. (a) y = 0 m. (b) y = 10 m. (c) y = 20 m. (d) y = 30 m.

Based on the above research, this study changes the depth of the calculation points and the distance between the cutterhead and the enclosure structure, and investigates their impact on the additional load. The depth variation range considered is z = 0–30 m, and the distances between the side wall of the receiving well and the cutterhead are set to 5 m, 10 m, 20 m, and 30 m, respectively. The additional load caused by the bulkhead additional thrust, the force of friction between the shield and soil, and the additional grouting pressure at specific *y*-values (y = 0m, y = 10m, y = 20m, and y = 30m) are calculated and illustrated in Figs 13–15.

Fig 14 illustrates the variation of the additional load in the *x*-direction caused by the bulkhead additional thrust along the depth. In Fig 14(A), the additional load in the *x*-direction at *y* = 0 is presented. The additional load initially increases and reaches its maximum value, then gradually decreases as the depth increases. The maximum value occurs at H = 13m, which corresponds to the center point of the cutterhead, with an approximate value of 2300 kPa. The additional load in the *x*-direction experiences a significant increase as the distance between the cutterhead and the enclosure structure decreases. Conversely, it decreases notably as the distance from the tunnel axis increases. When the distance from the tunnel axis exceeds 10m, the additional load becomes greater as the shield machine moves further away from the enclosure structure.

Fig 15 depicts the variation of additional load in the x-direction along the depth direction at *y* = 0 m, *y* = 5 m, *y* = 10 m, and *y* = 20 m respectively by the force of friction between shield and soil. The distribution of the additional load along the depth follows a similar pattern to that caused by the bulkhead additional thrust. The maximum value of the additional load occurs at the center point of the cutterhead. However, at y = 0m, there is a mutation in the distribution of the additional load along the depth when the distance between the cutterhead and the enclosure structure is 5m. This can be attributed to the size effect of the cutterhead compared to the length of the enclosure structure and their distance. When the distance between the cutterhead and the enclosure structure is relatively large, the influence of the frictional resistance of the shield shell on both sides on the center point can be neglected. Conversely, when the distance is too small, the superposition effect of the shield shells on both sides on the center point cannot be ignored. It is noteworthy that the additional load caused by the force of friction between the shield and soil is significantly higher than that caused by the bulkhead additional thrust.

**Fig 16:**
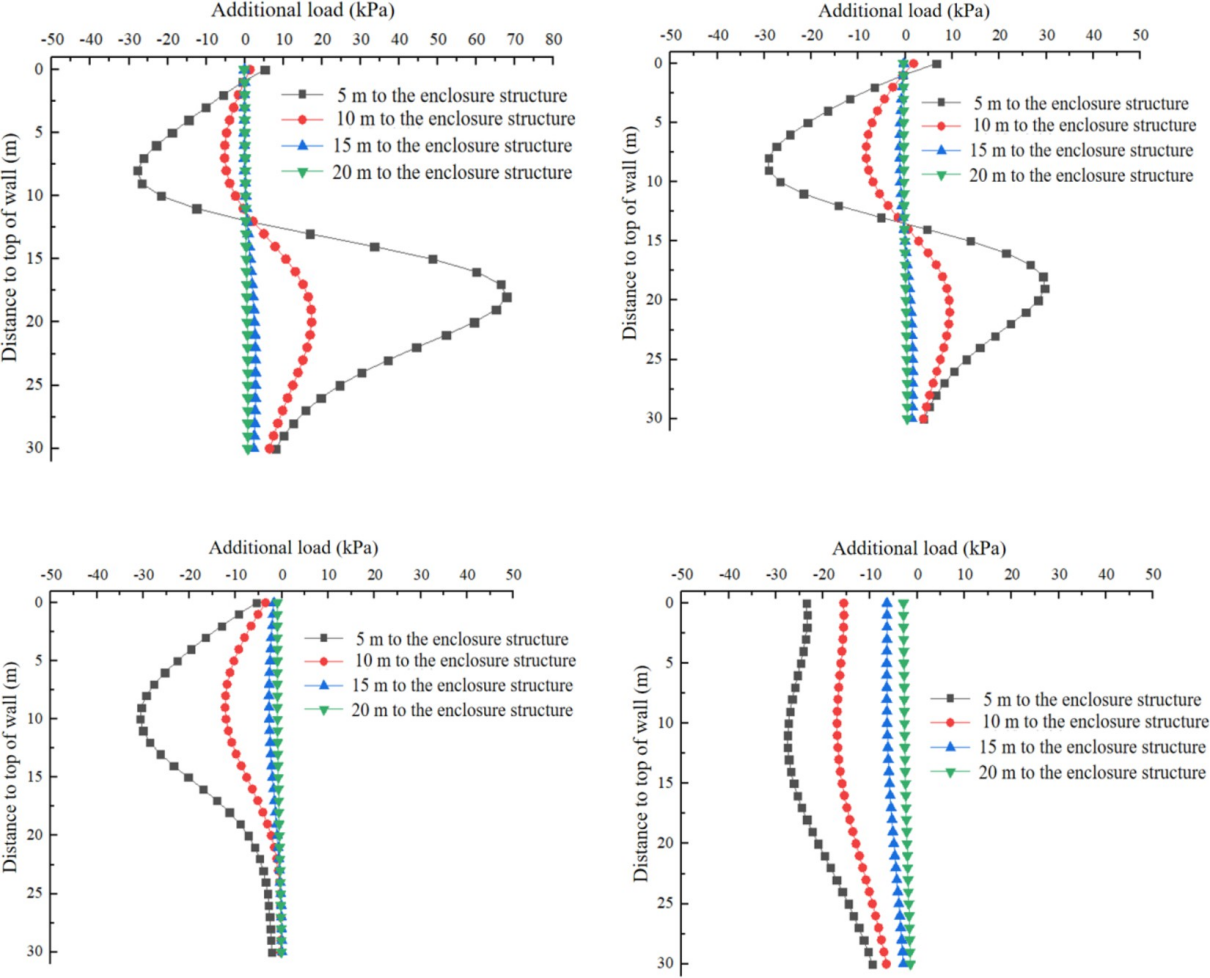
Additional load caused by additional grouting pressure. (a) y = 0 m. (b) y = 10 m. (c) y = 20 m. (d) y = 30 m.

Fig 16 shows the variation of additional load in the x-direction along the depth direction at *y* = 0 m, *y* = 5 m, *y* = 10 m, and *y* = 20 m respectively by additional grouting pressure. When *y* is within 10m, the additional load exhibits an antisymmetric distribution, with the center point of symmetry at the center point of the cutterhead. As *y* exceeds 10m, the additional load caused by the additional grouting pressure initially increases and then decreases with the increase of depth, and the direction of the additional load is in the *x*-negative direction. The additional load resulting from the additional grouting pressure increases as the distance between the cutterhead and the enclosure structure decreases, and this trend remains consistent regardless of the increase in *y*.

## Conclusion

In addressing the issue of additional stress and deformation in the receiving well caused by shield tunneling, a theoretical prediction formula for additional stress and deformation was derived using the two-stage method. Additionally, a numerical simulation model was developed based on an actual engineering case to analyze the deformation and additional load of the receiving well. The results of the research are as follows:

1. The calculation errors of the theoretical prediction results, numerical simulation results, and on-site monitoring data are relatively small, confirming the accuracy of the theoretical prediction formula and numerical simulation model.
2. As the distance between the shield machine and the receiving well decreases, the maximum deformation of the receiving well increases. Specifically, when the distance is 20m, 10m, and 5m, the maximum deformations are 0.8mm, 11.4mm, and 18.3mm, respectively. The proximity of the shield machine to the receiving well results in a significant increase in disturbance. When the shield machine is three times the length of the shield shell away from the receiving well, it is necessary to enhance monitoring of the docking well.
3. The additional load and deformation caused by bulkhead additional thrust and the force of friction between the shield and soil initially increase and then decrease along the depth. When the tunnel surface is close to the enclosure structure, there is a mutation in the additional stress and deformation at the center of the cutterhead. The deformation of the receiving well enclosure structure along the depth exhibits an antisymmetric pattern due to the additional grouting pressure, with the symmetric center at the center point of the cutterhead.
4. The primary factor contributing to the deformation of the receiving well enclosure structure is the force of friction between the shield and soil, followed by bulkhead additional thrust. The deformation caused by the additional grouting pressure near the shield tail can be disregarded.

In this paper, we do not address the scenario of receiving well reserved openings. These reserved openings can be considered as areas where the stiffness of the receiving well is reduced. We will conduct an analysis of the case involving receiving well reserved openings in a subsequent study.

## Supporting information

S1 Data (XLSX)

## References

[1] Jallow A., Ou C.Y., Lim A., Three-dimensional numerical study of long-term settlement induced in shield tunneling, Tunnelling and underground space technology. 2019, 88, 221–236. doi: 10.1016/j.tust.2019.02.021

[2] Guo X., Spatiotemporal Deformation of Existing Pipeline Due to New Shield Tunnelling Parallel Beneath Considering Construction Process, Applied Sciences. 2022, 12, doi: 10.3390/app12010500

[3] Do N.A., Dias D., Vu T.T., Dang V.K., Impact of the shield machine’s performance parameters on the tunnel lining behaviour and settlements, 2021, 10.1007/s12665-021-09820-2.

[4] Lou P., Li Y., Xiao H., Zhang Z., Lu S., Influence of Small Radius Curve Shield Tunneling on Settlement of Ground Surface and Mechanical Properties of Surrounding Rock and Segment, Applied Sciences. 2022, 12, 9119.

[5] Komiya K., Soga K., Akagi H., Hagiwara T., Bolton M., Finite element modelling of excavation and advancement processes of a shield tunnelling machine, Soils & Foundations. 2008, 39, 37–52. 10.3208/sandf.39.3_37.

[6] Kim K., Kim J., Ryu H., Rehman H., Jafri T.H., Yoo H., et al., Estimation Method for TBM Cutterhead Drive Design Based on Full-Scale Tunneling Tests for Application in Utility Tunnels, Applied Sciences. 2020, 10, 5187.

[7] Deng H.-S., Fu H.-L., Yue S., Huang Z., Zhao Y.-Y., Ground loss model for analyzing shield tunneling-induced surface settlement along curve sections, Tunnelling and Underground Space Technology. 2022, 119, 10.1016/j.tust.2021.104250.

[8] Wei Z., Jiang Y., A Simplified Analysis Method for the Deformation Response of an Existing Tunnel to Ground Surcharge Based on the Pasternak Model, Applied Sciences. 2021, 11, 3255.

[9] Liu B., Yu Z., Han Y., Wang Z., Wang S., Analytical solution for the response of an existing tunnel induced by above-crossing shield tunneling, Computers and Geotechnics. 2020, 124, 103624. 10.1016/j.compgeo.2020.103624.

[10] Lou P., Huang W., Huang X., Analysis of Shield Tunnels Undercrossing an Existing Building and Tunnel Reinforcement Measures, Applied Sciences. 2023, 13, 5729.

[11] Zhong-Hui Huang, Yong-Mao Hou, Ying-Nan Ren, Environmental Effect and Control of Large Diameter EPB Shield Tunneling below an Operating Airport, Journal of Aerospace Engineering. 2015, 10.1061/(ASCE)AS.1943-5525.0000446.

[12] Zhe W., Zhang K.W., Gang W., Li B., Qiang L., Yao W.J., Field measurement analysis of the influence of double shield tunnel construction on reinforced bridge, Tunnelling and Underground Space Technology. 2018, 81, 252–264. 10.1016/j.tust.2018.06.018.

[13] Alsahly A., Stascheit J., Meschke G., Advanced finite element modeling of excavation and advancement processes in mechanized tunneling, Advances in Engineering Software. 2016, 100, 198–214. 10.1016/j.advengsoft.2016.07.011.

[14] Huynh T.N., Chen J., Sugimoto M., Analysis on shield operational parameters to steer articulated shield, Japanese Geotechnical Society Special Publication. 2016, 2, 1497–1500. 10.3208/jgssp.ATC6-11.

[15] Miliziano S., de Lillis A., Predicted and observed settlements induced by the mechanized tunnel excavation of metro line C near S. Giovanni station in Rome, Tunnelling and Underground Space Technology. 2019, 86, 236–246. 10.1016/j.tust.2019.01.022.

[16] Nematollahi M., Dias D., Interaction between an underground parking and twin tunnels–Case of the Shiraz subway line, Tunnelling and Underground Space Technology. 2020, 95, 10.1016/j.tust.2019.103150.

[17] Hirai H., Settlements and stresses of multi‐layered grounds and improved grounds by equivalent elastic method, International Journal for Numerical & Analytical Methods in Geomechanics. 2010, 32, 523–557.

[18] Deng H., Fu H., Shi Y., Huang Z., Huang Q., Analysis of Asymmetrical Deformation of Surface and Oblique Pipeline Caused by Shield Tunneling along Curved Section, Symmetry. 2021, 13, 10.3390/sym13122396.

[19] Zhou Z., Chen Y., Liu Z., Miao L., Theoretical prediction model for deformations caused by construction of new tunnels undercrossing existing tunnels based on the equivalent layered method, Computers and Geotechnics. 2020, 123, 10.1016/j.compgeo.2020.103565.

[20] Zhang Z., Huang M., Boundary element model for analysis of the mechanical behavior of existing pipelines subjected to tunneling-induced deformations, Computers and Geotechnics. 2012, 46, 93–103. 10.1016/j.compgeo.2012.06.001.

[21] Mindlin R., Force at a Point in the Interior of a Semi‐Infinite Solid, Physics. 1936, 7, 195–202. 10.1063/1.1745385.

[22] Huang Z., Zhang H., Fu H., Ma S., Liu Y., Deformation Response Induced by Surcharge Loading above Shallow Shield Tunnels in Soft Soil, KSCE Journal of Civil Engineering. 2020, 24, 2533–2545. 10.1007/s12205-020-0404-8.

[23] Shivaei S., Hataf N., Pirastehfar K., 3D numerical investigation of the coupled interaction behavior between mechanized twin tunnels and groundwater–A case study: Shiraz metro line 2, Tunnelling and Underground Space Technology. 2020, 103, 10.1016/j.tust.2020.103458.

[24] Zhang M., Li S., Li P., Numerical analysis of ground displacement and segmental stress and influence of yaw excavation loadings for a curved shield tunnel, Computers and Geotechnics. 2020, 118, 10.1016/j.compgeo.2019.103325.

[25] Li M.-G., Zhang Z.-J., Chen J.-J., Wang J.-H., Xu A.-J., Zoned and staged construction of an underground complex in Shanghai soft clay, Tunnelling and Underground Space Technology. 2017, 67, 187–200. 10.1016/j.tust.2017.04.016.

[26] Zhou H.Y., Li L.X., Chen T.G., An Approach to Determine the Stiffness Reduction Factor of Tunnel Lining, Advanced Materials Research. 2011, 243–249, 3659–3662. 10.4028/www.scientific.net/AMR.243-249.3659.

